# Geriatric medicine and old age psychiatry joint training pre-feasibility pilot study: an innovative approach to collaborative postgraduate training

**DOI:** 10.1186/s12909-019-1716-6

**Published:** 2019-07-25

**Authors:** Carly Welch, Ayesha Bangash, Robert Wears, David Rice, Victor Aziz, Geriatric Medicine Research Collaborative, Lauren McCluskey, Qusai Bharmal, John Blair, Kadim Al-Fartoosi, Rebecca Stubbs, Holly Jacques, Farzad Khalkhali, Megan Offer

**Affiliations:** 1 Sandwell and West Birmingham Hospitals NHS Trust, Sandwell General Hospital, Lyndon, West Bromwich, West Midlands, B71 4HJ UK; 2 Institute of Inflammation and Ageing, College of Medical and Dental Sciences, University of Birmingham, Edgbaston, Birmingham, B15 2TT UK; 3 Health Education England (West Midlands), 213 Hagley Road, Edgbaston, Birmingham, B16 9RG UK; 4 South West Yorkshire Partnership NHS Foundation Trust, Fieldhead, Ouchthorpe Lane, Wakefield, WF1 3SP UK; 5 University Hospitals Birmingham NHS Foundation Trust, Solihull Hospital, Lode Lane, Solihull, B91 2JL UK; 6 British Geriatrics Society Education & Training Committee, Marjory Warren House, 31 St. John’s Square, London, EC1M 4DN UK; 7 Midlands Partnership NHS Foundation Trust, St. George’s Hospital, Corporation Street, Stafford, ST16 3SR UK; 8 Faculty of Old Age Psychiatry, Royal College of Psychiatrists, 21 Prescot Street, London, E1 8BB UK

**Keywords:** Collaboration, Geriatrics, Psychiatry, Education, Postgraduate, Training

## Abstract

**Background:**

Physical and psychological health problems are prevalent in older adults and rarely exist in isolation. Treating these problems in isolation is resourceful and can be potentially harmful to patients due to delays in diagnosis and treatment and incomplete holistic care plans. Historically, trainees in geriatric medicine and old age psychiatry within the United Kingdom have completed very different training programmes.

**Methods:**

We undertook a pre-feasibility pilot study of collaborative postgraduate training between trainees in geriatric medicine and old age psychiatry within the West Midlands training region, United Kingdom. Trainees in each specialty were paired with each other and advised to arrange appropriate training opportunities for their counterpart; these included shadowing each other in their workplace and arranging opportunities to attend training opportunities with their consultants. Pre- and post-pilot surveys were completed by all trainees and reflections from trainees were collated.

**Results:**

Five trainee pairs were formed and arranged shadowing and training opportunities between October 2017 and May 2018. This included a combination of inpatient, outpatient, and community work. For both specialties, trainees’ confidence in topics relating to their counterparts’ specialty increased between the pre- and post-pilot surveys. Recurrent themes included within reflections included the benefits of collaborative training.

**Conclusions:**

Our pilot has demonstrated that it is feasible to implement a programme of joint training into postgraduate medical training, and that this can have a positive impact upon the confidence of both specialties. An extended pilot is planned for the training year 2018–2019.

**Electronic supplementary material:**

The online version of this article (10.1186/s12909-019-1716-6) contains supplementary material, which is available to authorized users.

## Background

Over the last 50 years, life expectancy within the UK has continued to increase, leading to an increasing ageing population. However, increase in total lifespan has not been associated with increase in healthy lifespan [1]. Older adults have complex health and social care needs. Both physical and psychological health problems are prevalent amongst older adults, and these conditions rarely exist in isolation; multimorbidity is prevalent amongst older adults [2]. Biological, psychological, and social problems can all interact in affecting an older person’s health and wellbeing. Treating any of these problems in isolation can result in ineffectual management of the person’s needs, and increased demands to health and social care in the longer term [3].

Within the UK, doctors wishing to undertake careers in either geriatric medicine or old age psychiatry are required to complete very different training programmes. However, there is significant overlap of the skills and expertise that are required for both specialties. Whilst specific competencies in psychiatric problems of older age are requirements for completion of geriatric medicine training, these are sufficiently broad that training experience can vary significantly. Trainees in geriatric medicine are expected obtain knowledge of major psychiatric conditions and illnesses, clinical pharmacology for older people with psychiatric conditions, and psychiatric assessment methods and tools [4]. In addition, most training is organised on an ad hoc basis, with an emphasis upon the trainee themselves to make these arrangements. The old age psychiatry curriculum includes the recommendation that trainees should maintain their skills in assessment of physical health problems, but there is little reference to specific geriatric medicine competencies [5]. A previous survey of curriculum and training opportunities for old age psychiatry trainees within the UK demonstrated that both trainee and trainer respondents considered that the curriculum should include geriatric medicine competencies [6]. The old age psychiatry curriculum is expected to undergo revisions by the end of 2020, which are likely to include greater physical health competencies, in order to ensure standards set by the General Medical Council and recommendations of the Shape of Training review are met [7]. To date, there have been no formal recommendations upon how this training should be arranged. Interdisciplinary collaborative training offers an innovative approach to postgraduate training. We describe our initial pilot of a new geriatric medicine and old age psychiatry collaborative training programme.

## Methods

### Aim

To establish the feasibility of embedding a collaborative training programme within postgraduate geriatric medicine and old age psychiatry training in the UK.

### Design and setting

Higher specialist trainees in geriatric medicine and old age psychiatry within Health Education England (West Midlands) (HEE(WM)) were asked to volunteer to participate in this study of a new pilot training programme. Trainees were then paired up geographically. All trainees who participated in the study met with their regional Training Programme Directors (TPDs) and representatives of the Royal College of Psychiatry (RCPsych) Faculty of Old Age Psychiatry and British Geriatrics Society (BGS) at the start and throughout the course of the study. A pre-pilot survey (Additional file 1) was supplied to all trainees and requested to be completed prior to undertaking any collaborative training. Between October 2017 – May 2018, trainee pairs arranged to observe each other in their clinical roles, and to attend training activities arranged by the other trainee. Activities that trainees arranged for each other were ones that had been identified as relevant to a specific learning need by the trainees themselves, but the clinical practice that could be observed was broad. When conducting shadowing, trainees would not be involved in clinical decision making of patients, but would introduce themselves and explain their role at each encounter. Trainees would then subsequently discuss learning points from each case between them away from patients. A post-pilot survey (Additional file 2) was completed by trainees at the end of the study and informal feedback was relayed amongst the group. Both the pre-pilot and post-pilot surveys included 18 Likert-style questions, which required the trainees to rank their confidence in individual statements relating to geriatric medicine and old age psychiatry curriculum items (1 = not confident at all; 5 = very confident).

### Assessment of feasibility

Feasibility was defined as the completion of training experiences within their trainee counterpart’s specialty. We aimed to assess the proportion of trainee pairs who were able to undertake such training. The assessment of change in trainee confidence within curriculum items relating to their counterpart’s specialty, and qualitative data suggesting perceived increased knowledge or confidence formed secondary aspects of our feasibility analysis.

### Qualitative analysis

Trainees completed reflections following each training or learning event. These were collated alongside white space survey responses and assessed for recurrent themes in a structured manner using a semi-deductive approach. Text was initially screened for recurrent themes. Text was then reanalysed in detail and separate to match initial emerging themes; additional themes were added throughout this process as necessary. All collated information was then reviewed for important and most frequently occurring themes. Following completion of this pilot training programme, consultants who observed and supervised interspecialty training were contacted for anonymous retrospective feedback; this has been analysed and presented separately.

### Statistical analysis

Likert scale questions were organised into pertaining most closely to old age psychiatry (six questions), geriatric medicine (eight questions), or both specialties (four questions) (Table 1). The pre-pilot and post-pilot scores for all questions within each group were collated for geriatric medicine and old age psychiatry respectively. The median test scores for each group for each specialty were calculated and significance of differences between pre-pilot and post-pilot scores were assessed using Wilcoxon signed rank sum tests using IBM SPSS Statistics 22.

**Table 1:** Curriculum items assessed using Likert-style questions for each specialty. The pre- and post-pilot questionnaires both asked trainees to rate their confidence in these curriculum items. Out of 18 curriculum items, 6 were considered to relate to old age psychiatry, 8 to geriatric medicine, and 4 to both specialties

Old age psychiatry	Geriatric medicine	Both
1. Assessment and management of common psychiatric conditions 2. Pharmacology and non-pharmacological management in psychiatry 3. Behavioural and Psychotic Symptoms of Dementia 4. Differentiation between dementia, delirium, depression, dysphasia 5. Cognitive and mood assessment 6. Consider family, cultural, religious background in management of psychiatric health	1. Models and concepts of frailty 2. Diagnosis and management of delirium, falls, incontinence 3. Ethical decisions such as life-prolonging treatment, resuscitation, consent 4. Assessment of overall physical health, and arranging appropriate investigations 5. Recognition of acute illness 6. Functional status evaluation 7. Assisting and guiding core trainees in managing physical illness 8. Know when to refer to other specialists for physical health conditions	1. Assessment of mental capacity 2. Safeguarding 3. Managing long-term conditions in collaboration with other specialists 4. Communicating with patients from diverse backgrounds, and/or with sensory impairment

## Results

Twelve geriatric medicine trainees and four old age psychiatry trainees initially volunteered to participate. Four pairs were formed initially; a fifth was formed after another old age psychiatry trainee who later joined the rotation agreed to participate. The geriatric medicine trainees who were selected to participate were those who were working most closely to the five old age psychiatry trainees that volunteered. Geriatric medicine trainees ranged from Specialist Training years 3 to 7 (ST3-ST7) and old age psychiatry trainees ranged from ST4-ST6; this represents the full complement of training grades for both specialties (geriatric medicine is a five year training programme after two years of core training, whereas old age psychiatry is a four year training programme after three years of core training). Pre- and post-pilot surveys were returned from all trainees who participated. Pre-pilot surveys were returned by all geriatric medicine trainees prior to completion of the study; pre-pilot surveys were collected from old age psychiatry trainees following completion of the study and one of these included text written in the past tense about the pilot itself. Detailed reflections were returned from three of the geriatric medicine trainees and brief reflections were returned from all the old age psychiatry trainees. Additional feedback was provided from two consultants who observed or supervised trainee experiences.

In the pre-pilot surveys, none of the trainees expressed that they had had previous experience of working in a joint geriatric medicine and old age psychiatry unit. Ten out of the 12 trainees stated that they felt patients would “very much” benefit from access to jointly delivered services in the pre-pilot survey; this increased to all trainees in the post-pilot survey. Considering the Likert-style questions, there was one omission by a single geriatric medicine trainee in both the pre- and post-pilot surveys. There were no omissions by old age psychiatry trainees in the pre-pilot survey, although there were three omissions by old age psychiatry trainees in the post-pilot survey.

Trainees observed clinical practice in a variety of different settings during this study. The specific details of the shadowing opportunities that trainees undertook were not formally collated from surveys. However, this information was discussed at the final group meeting at the end of this pilot. Trainees in old age psychiatry were able to observe review of patients referred by other specialties (orthopaedics, general surgery), review of patients on geriatric medicine ward rounds, review of patients with strokes, or stroke-like presentations in the emergency department, and attend geriatric medicine outpatient clinics. Some of these patients had psychiatric disturbances such as delirium, dementia, or depression, but psychiatric trainees were importantly able to revise their knowledge of physical health conditions. Trainees in geriatric medicine were able to observe review of patients with cognitive impairment in memory clinic, psychotherapy sessions, complex home visits, and attend inpatient and complex care units. This included, but was not limited to, observation of new diagnosis of dementia, dose titration of acetylcholinesterase inhibitors, and management of Behavioural and Psychotic Symptoms of Dementia.

### Quantitative results

Results of the Likert-style questions are displayed in Table 2. The median score in the pre-pilot was higher for geriatric medicine competency questions amongst geriatric trainees and higher for old age psychiatry competency questions amongst old age psychiatry questions. The median for questions pertaining to both specialties was higher amongst geriatric trainees. The median score for geriatric medicine and both specialties competency questions improved between the pre-pilot and post-pilot for both geriatric medicine trainees and old age psychiatry trainees. Median score for old age psychiatry competency questions improved for geriatric medicine trainees but not old age psychiatry trainees.

**Table 2:** Median test scores for both geriatric medicine and old age psychiatry trainees for each specialty group of questions. Confidence in old age psychiatry competencies increased between pre- and post-pilot surveys amongst geriatric medicine trainees; confidence in geriatric medicine competencies increased between pre- and post-pilot surveys amongst old age psychiatry trainees. *P*-value calculated using related samples Wilcoxon signed rank sum test

	Geriatric medicine			Old age psychiatry		
	Pre-pilot (median score)	Post-pilot (median score)	*p*-value	Pre-pilot (median score)	Post-pilot (median score)	*p*-value
Geriatric medicine competency questions	4	5	< 0.001	3	4	< 0.001
Old age psychiatry competency questions	3	4	< 0.001	4	4	0.080
Competency questions pertaining to both specialties	4	5	< 0.001	3	4	0.001

### Qualitative results

#### Collaborative working

Evaluation of reflections and white space questions of post-pilot surveys revealed a common theme emerging that both geriatric medicine trainees and old age psychiatry trainees considered that there were benefits to collaborative working.

*“Attendance at this clinic demonstrated the potential benefit of collaborative working between geriatricians and old age psychiatrists.” (Geriatric trainee, ST5)*

*“There are gross similarities between the patients and problems that both specialties face, but both have a slightly different focus” (Geriatric trainee, ST5)*

*“My team found his input quite beneficial and [his] team felt the same when I shadowed him.” (Old age psychiatry trainee, ST5)*

*“Patients are better managed when there are good links between specialties.” (Old age psychiatry trainee, ST5)*

#### Learning achieved by geriatric medicine trainees

Common themes that emerged from reflections and white space questions supplied by geriatric medicine trainees included increased knowledge of pharmacology and prescribing for older adults with psychiatric conditions, increased confidence in assessment and management of cognitive spectrum disorders, and greater appreciation of the benefits and complexities of community assessment.

*“It was particularly interesting to learn of the infrequency of which psychiatrists prescribed sedative medication for management of agitation …. I felt that members of the MDT were generally more comfortable in managing such symptoms than staff within secondary care and that there was less pressure to prescribe sedative medication for management of complex patients.” (Geriatric medicine trainee, ST5)*

*“It was interesting to observe acetylcholinesterase inhibitors being commenced and dose adjusted dependent on patient presentation.” (Geriatric medicine trainee, ST5)*

*“Doses of acetylcholinesterase inhibitors can be escalated when symptoms of [Behavioural and Psychotic Symptoms of Dementia (BPSD)] exist rather than commencing antipsychotics” (Geriatric medicine trainee, ST5)*

*“This case has allowed me to reflect on my own practice and considered that a positive diagnosis of dementia should be given early where appropriate.” (Geriatric medicine trainee, ST5)*

*“Although dementia diagnosis is often referred to old age psychiatry, the process is no different to that encountered by geriatricians and geriatricians should feel more confident (and should be supported) in making these diagnoses as well” (Geriatric medicine trainee, ST5)*

*“It was interesting to see the different dynamics of a home visit compared to a clinical setting. It highlighted how complex elderly psychiatry needs can be, especially when two people in a household are affected by mental health issues.” (Geriatric medicine trainee, ST5)*

*“It also made me appreciate the down side of a home visit when the environment is distracting and you do not have all of the patient’s previous records at hand.” (Geriatric medicine trainee, ST5)*

#### Learning achieved by old age psychiatry trainees

Common themes that emerged from reflections and white space questions supplied by old age psychiatry trainees included the value of revising their knowledge of assessment and management of common physical health problems and increased understanding of frailty.

*“I found this experience useful in refreshing my skills in physical health in old age.” (Old age psychiatry trainee, ST6)*

*“One tends to forget stroke mimics when working in psychiatry however it is common to see suspected stroke cases. I found this experience useful in brushing up one's skills in managing physical health skills.” (Old age psychiatry trainee, ST6)*

*“When working in old age one tends to forget all causes of fevers in the elderly.” (Old age psychiatry trainee, ST6)*

*“I was surprised to know that elderly patients with significant co-morbidities can undergo major operations.” (Old age psychiatry trainee, ST4)*

*“I liked the practice of screening all patients in geriatric settings for certain conditions which can be referred to throughout their admission e.g. delirium screening.” (Old age psychiatry trainee, ST5)*

#### Feedback from consultants

Consultants who observed or supervised interspecialty training activities, and were willing to provide anonymous feedback provided positive feedback on the pilot.

*“An interesting piece of work ….. very useful for the psychiatry trainees to get involved in gaining experience in geriatric medicine and vice versa.” (Consultant Old Age Psychiatrist)*

*“I personally and professionally found the whole process hugely beneficial. We found there was more overlap between our roles than either party had realised. I really enjoyed being able to teach on a subject I’m passionate about and also took great learning from having the [geriatric] SPR shadow me. The teams I work for also found it really helpful to have an opportunity to ask some medical queries about common [problems] that we come across regularly but perhaps don’t feel that confident in managing.” (Consultant Old Age Psychiatrist)*

### Feasibility and barriers to implementation

All trainees who participated in this pilot were able to undertake some form of shadowing experience. However, many trainees initially expressed concern that they would not be able to find time to complete this training due to service requirements, and under-valuing of its importance by supervisors. In practice, supervisors were supportive of this activity, and no trainees were prevented from undertaking training due to service requirements. In the post-pilot survey, some trainees made the recommendation that the scheme should be made “mandatory” and “formalised”. Trainees were able to arrange their shadowing and observation activities within their individual pairs by liaising far in advance to find suitable days that did not conflict with service requirements, annual leave, or study leave; service requirements were generally not a reason to prevent a trainee shadowing them, providing this was compatible with their identified learning needs.

## Discussion

The results of the pre- and post-pilot surveys demonstrated that both specialties’ trainees’ confidence in curriculum items relating to the other trainees’ specialty improved following participation in this pilot. This suggests that participation in the pilot itself had a positive impact. However, it should also be noted that the confidence of geriatric trainees in curriculum items relating to their own specialty improved following participation in this pilot. The survey was designed to measure confidence rather than competence, and it is plausible that the confidence of geriatric medicine trainees in their abilities and understanding of their own specialty was improved through providing training for their old age psychiatry colleagues. However, this pilot was not conducted in isolation from other training and trainees in both specialties would have had continued training in their own specialty within this time. This is important to consider as it is possible that trainees from either specialty could have received training related to the other specialty elsewhere, although this was not declared by any of the trainees.

Considering that trainees should have obtained training in their own specialty elsewhere, it could be considered unexpected that there was no difference in the pre- and post-pilot scores for old age psychiatry trainees’ confidence in their own specialty. Both specialties include a mixture of trainee grades who participated, so this was unlikely to be related to the effect of seniority leading to knowledge saturation. It is important to consider that the Likert scale responses provided ordinal rather than continuous data, and the difference between a score of 3 and 4 could not be considered the same as the difference between 4 and 5. Trainees in old age psychiatry may have developed increased confidence in their own specialty insufficient to be detectable with this survey. In addition, although trainees in old age psychiatry would have been expected to develop some increased confidence in both geriatric medicine competencies and their own specialty within this timeframe, the rate of increase related to geriatric medicine would be expected to be far greater.

Unfortunately, pre-pilot survey responses for old age psychiatry trainees were collected following completion of the study itself, therefore, it is not possible to be certain of when these were completed. At least one of these surveys was written following completion of the pilot as white space questions were written in the past tense and referred to experiences that had been undertaken. This may have introduced recall bias into the responses that were given. However, all pre-pilot survey responses were collected from geriatric medicine trainees at the start of the pilot. Whilst we cannot be certain of the impact of recall bias upon responses for old age psychiatry trainees, we can be confident that this did not impact upon the responses from geriatric medicine trainees. Importantly, a similar improvement in median scores for confidence in each other’s specialties was demonstrated.

The quantitative analysis of survey responses corroborates with our qualitative analysis. Trainees appreciated that there was significant overlap between patients managed by geriatric medicine and old age psychiatry, but that the focus of their management differed. Trainees appreciated the potential benefits to patients of collaborative working, as well as the benefits to their training in undertaking this pilot. Geriatric medicine trainees were able to develop their confidence of diagnosing and managing cognitive spectrum disorders, with a particular focus on the prescription of acetylcholinesterase inhibitors. Old age psychiatry trainees were able to develop their knowledge of frailty and management of physical health problems. We did not record interviews of trainees who participated in this pilot and all qualitative data was obtained from survey responses or reflections. However, even within this small sample, common themes clearly emerged; although we cannot be certain if saturation was achieved with this sample size. Our qualitative analysis corroborates with discussion with trainees who met as a group at the end of the pilot, although this was not formally recorded.

Feedback provided from consultants is concordant with trainee feedback. This is important as self-perceived competence has been shown not to correlate with objectively measured confidence in other studies of medical education [8, 9]. However, this feedback is extremely limited due to the low responder bias, as well as recall bias; this feedback was obtained after the completion of the pilot. One of the old age psychiatry consultants who provided feedback described a positive benefit to their own professional development, in improving their own physical health knowledge; this was a positive secondary consequence of this pilot that had not been considered before. This emphasises the benefits of collaborative working between the two specialties, which we feel can be strengthened by a collaborative model of postgraduate training.

Importantly, we have shown that this model of collaborative training is potentially feasible. Within this initial sample, all trainees were able to access some training opportunities or experiences in their counterpart’s specialty. This pilot has demonstrated the importance of clear communication between trainee pairs and sufficient time available for careful planning of training arrangements. One of the most commonly expressed concerns by trainees in undertaking this pilot was being supported by their supervisors to take time away from their service commitments. The involvement of training programme directors and college leads can assist to enforce the importance of these training activities. We strongly encourage formal college involvement for similar training models in the future. The improvements in confidence scores for curriculum items that were measured suggest that trainees were able to access training opportunities that mapped with the curriculums. The positive comments of both collaborative training and working in general, and perceived learning within each specialty demonstrate that this collaborative training model can have a constructive impact upon the training of trainees in both specialties.

Although the results of this pilot are very promising, it should be considered that there were a small number of trainees who participated. Additionally, as trainees were asked to volunteer to participate, there may have been some bias in the level of engagement by trainees; trainees who volunteered may be more likely to engage than trainees who did not. We fully acknowledge that our results may not be representative of all trainees. A further larger study is planned to establish if this model of training is feasible at scale. This will involve all trainees in both specialties working within HEE(WM) who are currently in post. One of the anticipated difficulties is that there is a disparity in the number of trainees within each specialty (62 geriatric trainees vs 13 old age psychiatry trainees within HEE(WM) in the academic year 2017–18). In order to accommodate for this, in the next academic year the pilot will be undertaken with each psychiatric trainee paired with three to four geriatric medicine trainees. Trainee groups will be expected to meet together throughout the year to share learning experiences. Geriatric medicine trainees will each be given the opportunity to arrange training with the old age psychiatry trainee in their group; the old age psychiatry trainee will be able to arrange training with any or all of the geriatric medicine trainees. The results of this larger regional extended feasibility study will be used to develop national recommendations for joint training.

We consider that this model of training could be applied to other specialties with overlapping patient needs and curriculums, within both the UK, and other countries. This training model may have particular benefits in improving the efficiency of training different specialties in countries where training resources are limited. We are not aware of a similar training programme that has been undertaken elsewhere to date. Our larger scale study will help to greater inform specialties wishing to develop a similar model of collaborative training in the future. As an initial step when considering developing such a scheme, we would suggest meetings are arranged to include TPDs and trainee representatives for each specialty to decipher what the possible challenges to implementation may be, and how training needs of each specialty complement each other. Integration of geriatric medicine and old age psychiatry services as well as training is considered an innovative approach to addressing the healthcare needs of older adults [10]. Collaborative working amongst different specialties in general has demonstrated a positive impact upon patient care [11]. In addition, collaborative interdisciplinary training involving undergraduates in medicine, nursing, and allied health has demonstrated increased confidence amongst students [12, 13].

## Conclusions

It is feasible to embed a joint training programme into geriatric medicine and old age psychiatry postgraduate training. Older adults have complex health needs requiring both psychiatric and physical expertise. Interdisciplinary collaborative training between geriatric medicine and old age psychiatry trainees has the potential to improve the confidence of trainees in both specialties in managing both psychiatric and physical health problems. This has the potential to lead to long-term collaborative working between specialties.

## Additional files

Additional file 1: Pre-pilot survey. This file includes the initial version of the pre-pilot survey that was distributed to trainees prior to participation in this training pilot for completion. (DOCX 29 kb)

Additional file 2: Post-pilot survey. This file includes the post-pilot survey that was distributed to trainees following participation in this training pilot for completion. (DOCX 34 kb)

## Data Availability

The datasets used and analysed during the current study are available from the corresponding author on reasonable request.

## Abbreviations

BGS: British Geriatrics Society
HEE (WM): Health Education England (West Midlands)
MDT: Multidisciplinary Team
RCPysch: Royal College of Psychiatrists
TPD: Training Programme Director
