# Supplementary material for: Geriatric medicine and old age psychiatry joint training pre-feasibility pilot study: an innovative approach to collaborative postgraduate training
Source: BMC Med Educ. 2019 Jul 25;19:283. doi: 10.1186/s12909-019-1716-6 (PMC6659292; doi:10.1186/s12909-019-1716-6)
Supplement: Supplementary file 1 — Pre-pilot survey. This file includes the initial version of the pre-pilot survey that was distributed to trainees prior to participation in this training pilot for completion. (DOCX 29 kb) [file 12909_2019_1716_MOESM1_ESM.docx]

**"Integrated Old Age Training: A Collaboration between Psychiatrists and Geriatric Medicine”**

**Pre-Pilot Survey**

**Thank you for your interest in the Integrated Old Age Training Project. We would be very grateful if you would complete the following questionnaire in order to help guide and evaluate the pilot. Your responses will remain confidential.**

Top of Form

**1. To begin, please begin by entering your unique identifier code, in the following format:

INITIALS-year of birth    (for example, RC-1984)

Responses remain confidential. The code will be used to evaluate the initiative when you complete your post-pilot questionnaire**

**2. Enter your Specialty**

Geriatric Medicine

Old Age Psychiatry

Other (e.g. GP)

Other (please specify)

**3. Enter your Grade of Training**

ST1

ST2

ST3

ST4

ST5

ST6

ST7

ST8

**4. And the geographical area in which you are training: North Staffordshire Combined Healthcare Trust**

**5. Have you had any experience of working in a jointly delivered Geriatric Medicine and Old Age Mental Health Services?**

Yes

No

Bottom of Form

**6. How frequently do you currently have inter-professional communication with a doctor from your linked specialty?**

Daily

Weekly

Fortnightly

Monthly

Less frequently than once a month

**7. What proportion of these contacts are emergencies?**

<25%

25-50%

50-75%

75-100%

**8. To what extent does your current training enable you to achieve the competencies for your specialty as set out in the Integrated Old Age Training pilot information pack?**

I don't know

Not at all

Not really

Somewhat

Very much

**9. How confident do you feel in assessing and managing the following?**

|  | **Not confident at all 1** | **2** | **3** | **4** | **Very confident 5** |
| --- | --- | --- | --- | --- | --- |
| **ILO1** **To know how to assess and manage older patients presenting with the common psychiatric conditions** |  |  |  |  |  |
| **ILO2**  **Clinical Pharmacology, therapeutics and non-pharmacological management for psychiatric conditions** |  |  |  |  |  |
| **ILO3 Assessment of behavioural and psychological symptoms associated with dementia** |  |  |  |  |  |
| **ILO4 Differentiating between dementia, delirium and other diagnoses depression, dysphasia** |  |  |  |  |  |
| **ILO5**  **Cognitive and mood assessment** |  |  |  |  |  |
| **ILO6 10. To take account of a patient’s family, cultural and religious background bettering order to enable the management of the psychiatric health of the individual patient** |  |  |  |  |  |
| **ILO7 Assessment and documentation of mental capacity** |  |  |  |  |  |
| **ILO8**  **Factors influencing health status in older people and models and concepts of frailty** |  |  |  |  |  |
| **ILO9**  **Recognition, diagnosis and management of Delirium, Falls, Immobility, Incontinence (urinary and faecal)** |  |  |  |  |  |
| **ILO10 Be familiar with safeguarding legislation and actions needed when caring for vulnerable adults** |  |  |  |  |  |
| **ILO11** **Be familiar with ethical issues such as decisions regarding life-prolonging treatments, Resuscitation decisions, Consent procedures** |  |  |  |  |  |
| **ILO12**  **Assess the physical health of patients on admission and at appropriate intervals thereafter, including the assessment for the presence or absence of illness, injury or disability, and any of the following – receiving a medical history, making a functional enquiry, undertaking a physical examination, arranging blood tests and other investigations for the presence of an underlying physical cause, as necessary.** |  |  |  |  |  |
| **ILO13 Recognition of acute illness (the ‘deteriorating patient’): this includes measuring physiological parameters, using NEWS (National Early Warning Score) system, making the ‘first response’ to an acute illness, and using effective communication and resuscitation techniques.** |  |  |  |  |  |
| **ILO14 Monitor and provide treatment for long-term conditions in collaboration with specialists including the awareness of all medicines prescribed for the patient.** |  |  |  |  |  |
| **ILO15 Communicate effectively with patients from diverse backgrounds and those with special communication needs such as hearing, visual and speech impairments and confusion and mental health issues** |  |  |  |  |  |
| **ILO16 Functional status evaluation, including assessment of basic ADL and IADL, social support, mental health and cognitive status, mobility including gait and balance, and nutritional evaluation (assessment scales) used to assess functional status** |  |  |  |  |  |
| **ILO17** **Assist and guide Core trainees in assessing and managing patients with physical illness** |  |  |  |  |  |
| **ILO18 Know ‘when and how’ to refer to other health professionals. In particular, be aware of clinical ‘red flag’ symptoms and signs that should prompt referral to specialists and facilitate recovery from physical illness or injury for instance by liaison with specialists who provide the services needed by the patient for physical healthcare** |  |  |  |  |  |
|  |  |  |  |  |  |

**10. How well do you understand the nature and provision of services in your linked specialty (e.g. referral criteria and process, specialty clinics, emergency contacts) within the geographical area in which you work?**

Not at all

Not really

Somewhat

Very much

**11. To what extent do you feel that your patients would benefit from access to jointly delivered Geriatric Medicine and Old Age Mental Health services?**

Not at all

Not really

Undecided

Somewhat

Very much

**12. Please state 3 benefits you anticipate you will gain from involvement in the Integrated Old Age Training project**

**13. Please state any concerns you have about involvement in the Integrated Old Age Training Project**

**14. How did you hear about this initiative?**

From another trainee

From a consultant

Social media

Official college or deanery communication

Other (please specify)
